# Toxic Exposure to Endocrine Disruptors Worsens Parkinson’s Disease Progression through NRF2/HO-1 Alteration

**DOI:** 10.3390/biomedicines10051073

**Published:** 2022-05-05

**Authors:** Ramona D’Amico, Enrico Gugliandolo, Rosalba Siracusa, Marika Cordaro, Tiziana Genovese, Alessio Filippo Peritore, Rosalia Crupi, Livia Interdonato, Davide Di Paola, Salvatore Cuzzocrea, Roberta Fusco, Daniela Impellizzeri, Rosanna Di Paola

**Affiliations:** 1 Department of Chemical, Biological, Pharmaceutical and Environmental Sciences, University of Messina, 98166 Messina, Italy; rdamico@unime.it (R.D.); rsiracusa@unime.it (R.S.); tiziana.genovese@unime.it (T.G.); aperitore@unime.it (A.F.P.); interdonatol@unime.it (L.I.); dipaolad@unime.it (D.D.P.); dimpellizzeri@unime.it (D.I.); 2 Department of Veterinary Science, University of Messina, 98168 Messina, Italy; egugliaandolo@unime.it (E.G.); rcrupi@unime.it (R.C.); dipaolar@unime.it (R.D.P.); 3 Department of Biomedical, Dental and Morphological and Functional Imaging, University of Messina, 98125 Messina, Italy; marika.cordaro@unime.it; 4 Department of Clinical and Experimental Medicine, University of Messina, 98125 Messina, Italy

**Keywords:** endocrine disruptors, atrazine, Parkinson’s disease, inflammation, oxidative stress

## Abstract

Human exposure to endocrine disruptors (EDs) has attracted considerable attention in recent years. Different studies showed that ED exposure may exacerbate the deterioration of the nervous system’s dopaminergic capacity and cerebral inflammation, suggesting a promotion of neurodegeneration. In that regard, the aim of this research was to investigate the impact of ED exposure on the neuroinflammation and oxidative stress in an experimental model of Parkinson’s disease (PD). PD was induced by intraperitoneally injections of MPTP for a total dose of 80 mg/kg for each mouse. Mice were orally exposed to EDs, starting 24 h after the first MPTP administration and continuing through seven additional days. Our results showed that ED exposure raised the loss of TH and DAT induced by the administration of MPTP, as well as increased aggregation of α-synuclein, a key marker of PD. Additionally, oral exposure to EDs induced astrocytes and microglia activation that, in turn, exacerbates oxidative stress, perturbs the Nrf2 signaling pathway and activates the cascade of MAPKs. Finally, we performed behavioral tests to demonstrate that the alterations in the dopaminergic system also reflected behavioral and cognitive alterations. Importantly, these changes are more significant after exposure to atrazine compared to other EDs. The results from our study provide evidence that exposure to EDs may play a role in the development of PD; therefore, exposure to EDs should be limited.

## 1. Introduction

Human exposure to environmental pollutants such as endocrine disruptors (EDs) has attracted considerable attention in recent years as a result of epidemiological and experimental investigations linking them to altered human health [1]. EDs are found at low doses in thousands of products and can be classified into the following different groups: (i) food contact materials such as bisphenol A; (ii) chemicals in products such as diethyl phthalate (DEP) or perfluorooctanesulfonic acid (PFOS); (iii) synthetic estrogen such as 17α-ethinyl estradiol (EE) and (iv) pesticides such as cypermethrin (CP), vinclozolin (VCZ) and atrazine (ATR) [2]. Importantly, ATR is the most commonly found pesticide in surface and groundwater, where it tends to persist, as it has a half-life of 95–350 days and is resistant to degradation [3]. Most of the existing research concerns the effects of EDs on the reproductive system. However, recent data proposes that exposure to EDs produced abnormalities in behavior and brain functions [4,5]. Different studies showed that ED exposure may exacerbate the deterioration of the nervous system’s dopaminergic capacity and cerebral inflammation, suggesting a promotion of neurodegeneration [6,7]. Thus, it can be hypothesized that exposure to EDs could modify specific molecular mechanisms or signaling pathways, causing alterations in the structure and function of the Central Nervous System (CNS). However, to date, the mechanism of action for these EDs remains not fully understood. The most accredited hypothesis that could explain the mechanism of toxicity induced by ED exposure is the production of reactive oxygen species (ROS) that leads to an imbalance in the physiological antioxidant system involving nuclear factor-erythroid 2-related factor 2 (Nrf2) [8,9]. The response to oxidative stress also appears to be mediated in part by the activation of the MAPK cascade [10]. Excessive ROS formation has been listed as a major mechanism related to Parkinson’s disease (PD) and can trigger many types of damage, including neuroinflammation [11,12], characterized by the presence of activated microglia and reactive astrocytes in the brain parenchyma (indicated by the expression of Iba-1 and GFAP, respectively) and proinflammatory cytokines release. Neuroinflammation activation may quickly induce the degeneration of dopaminergic neurons in the midbrain [13], causing the alteration of dopamine (DA) production with related markers, such as tyrosine hydroxylase (TH) and dopamine transporter (DAT). The alteration of DA metabolism can lead to the accumulation of neurotoxic metabolites, which, in turn, can induce conformation changes of the endogenous α-synuclein (α-syn) protein which pathogenetically aggregates inclusion structures called Lewy bodies [14].

Considering the above, the rationale for this study was to investigate the neurotoxic effects of ED exposure during the development of PD and the potential cellular and molecular mechanisms underlying the disease. To do this, we used a mouse model based on the use of the neurotoxin 1-methyl-4-phenyl-1,2,3,6-tetrahydropyridine (MPTP) that induces biochemical and neuropathological changes as observed in human PD [15]. In addition to these parameters, we observed how these alterations affect behavioral deficits. The significance of the research is to clarify the mechanisms of ED-induced oxidative stress and neurotoxicity, which are important to emphasize the risk of human health following ED exposure and the pathogenesis of neurodegenerative disorders.

## 2. Materials and Methods

### 2.1. Animals

CD1 mice (male, 25–30 g, Envigo, Milan, Italy) were used in these studies. Mice were housed in individual cages (5 per cage) and posted in a controlled environment. The Review Board for the Care of Animals of the University of Messina approved the study. We respected the legislation for the protection of laboratory animals (D.Lgs 2014/26 and EU Directive 2010/63).

### 2.2. MPTP-Induced PD and Treatments

For MPTP intoxication, animals received four intraperitoneal (i.p.) injections of MPTP (20 mg/kg) in a saline solution at 2-h intervals in 1 day; the total dose was 80 mg/kg for each mouse.

Mice received by oral gavage the following EDs: CP (15 mg/kg), DEP (2 μg/mL), VCZ (100 mg/kg), EE (1 μg/kg), PFOS (10 mg/kg) and ATR (25 mg/kg). Exposure to EDs started 24 h after the first MPTP administration and continuing through 7 additional days after the last administration of MPTP. The dose of MPTP and the EDs used were based on previous in vivo studies and the literature [3,15,16,17,18,19,20,21].

### 2.3. Experimental Groups

Mice were randomly allocated into the following groups:

- MPTP: mice were subjected to the above-described PD induction;
- MPTP + CP: same as the MPTP group, and CP (15 mg/kg) was administered;
- MPTP + DEP: same as the MPTP group, and DEP (2 μg/mL) was administered;
- MPTP + VCZ: same as the MPTP group, and VCZ (100 mg/kg) was administered;
- MPTP + EE: same as the MPTP group, and EE (1 μg/kg) was administered;
- MPTP + PFOS: same as the MPTP group, and PFOS (10 mg/kg) was administered;
- MPTP + ATR: same as the MPTP group, and ATR (25 mg/kg) was administered;
- Sham groups = only saline solution was administered i.p. during the 1st day, such as how the MPTP protocol and vehicle or CP, or DEP or VLZ, or EE or PFOS or ATR were administered for 7 days.

Mice were sacrificed eight days after MPTP injection, and the brains were collected for histological and biochemical investigation.

### 2.4. Histological Analysis

Midbrain sections were stained with hematoxylin/eosin (H/E) using a Leica DM6 microscope associated with Leica LAS X Navigator software (Leica Microsystems SpA, Milan, Italy). For histological score, slides were scored for severity of the pathological profiles after H/E staining using a semiquantitative 5-point rating scale: 0 = normal, no death neuron observed; 1 = slight pathology, one to five death neurons; 2 = modest pathology, five to 10 death neurons; 3 = severe pathology, more than 10 death neurons and 4 = more severe pathology, only death neuron [22].

### 2.5. Immunohistochemical Analysis of TH and α-syn

An immunohistochemical analysis was performed as previously described [23]. Sections of the midbrain were incubated with the primary antibodies: anti-TH (Millipore, Milan, Italy) and anti-α-syn (Santa Cruz Biotechnology (SCB), Milan, Italy). Images were collected using a Leica DM6 microscope (Leica Microsystems SpA, Milan, Italy) following a typical procedure. The histogram profile is related to the positive pixel intensity value obtained.

### 2.6. Western Blot Analysis of TH, DAT, α-syn, GFAP, Iba-1, Nrf-2, Heme Oxigenase-1 (HO-1) and Neuronal Nitric Oxide Synthase (nNOS), p-JNK, p-ERK 1/2 and p-p38

Western blot analysis was performed on the midbrain samples as previously described [24]. The following primary antibodies were used: anti-TH (Millipore), anti-DAT (SCB, sc-32258), anti-α-syn (SCB, sc-32280), anti-GFAP (Novus Biologicals, Milan, Italy), anti-Iba1 (Abcam, CA, USA), anti-Nrf2 (SCB, sc365949), anti-HO-1 (SCB, sc-390991), anti-nNOS (Cell Signaling Technology (CST), Heidelberg, Germany), anti-p-JNK (CST), anti-p-p38 (CST), anti-p-ERK1/2 (SCB, sc7383), anti-α-actin (SCB, #sc8432) and anti-Lamin A/C antibody (Sigma-Aldrich, St. Louis, MO, USA). Protein expression was quantified by densitometry with BIORAD ChemiDoc™ XRS+ software and normalized to housekeeping genes α-actin and Lamin A/C, as previously reported [25].

### 2.7. Enzyme-Linked Immunosorbent Assay (ELISA)

The supernatant of the homogenate of the midbrain tissues was centrifuged (14,000 rpm at 4 °C for 30 min) and operated [26,27,28]. Levels of superoxide dismutase (SOD), catalase (CAT), glutathione (GSH-Px) system, tumor necrosis factor-α (TNF-α), interleukin (IL)-6 and IL-1β were measured using ELISA kits (R&D Systems, Minneapolis, MN, USA). The manufacturer’s manuals provided specific instructions for the test procedure. The absorbance value of each well was measured at 450 nm by a microplate reader.

### 2.8. Measurement of ROS Formation

ROS generation in the midbrain was evaluated by using 2′,7′-dichlorofluorescin diacetate (DCFH-DA; Sigma-Aldrich Corp., Milan, Italy), as previously described [29]. The ROS levels were represented as the fluorescence intensity and normalized to the sample protein. Fluorescence was measured at an excitation wavelength of 485 nm and an emission wavelength of 530 nm using a fluorescence microplate reader.

### 2.9. Evaluation of Lipid Peroxidation

The malondialdehyde (MDA) levels were quantified in the midbrain tissue to detect lipid peroxidation, as previously reported [30]. Briefly, midbrain tissues were homogenized in 1.15% KCl solution. An aliquot of the homogenate was added to a reaction mixture containing sodium dodecyl sulfate (SDS), acetic acid (pH 3.5), thiobarbituric acid and distilled water. Samples were then boiled, and MDA absorbances was detected at 650 nm using a spectrophotometer [31]. Levels were expressed in μmol/mg protein.

### 2.10. Nitrite/Nitrate Assay

The nitrite and nitrate levels were measured in the midbrain by a nitrate/nitrite colorimetric assay kit (Cayman Chemical-780001, Ann Arbor, MI, USA) according to the manufacturer’s instructions. Briefly, the tissues were homogenized in PBS buffer and centrifuged at 10,000× *g* for 20 min at 4 °C. Tissue level nitrite / nitrate analyzes were performed via Greiss reaction. Nitrate cannot be measured directly. In this method, the nitrate in the SN was converted into nitrite by nitrate reductase, and the total nitrite levels were determined as the total nitrate/nitrite (NOx) [30,32].

### 2.11. Behavioral Test

Mice were subjected to behavioral tests on days 0 and 8 days after MPTP injection. The data at time point 0 were not shown, as no significant differences between the different groups were observed. The mice were placed in behavior rooms 5 min for 2 days for acclimation prior to the start of behavioral testing. Mice were transferred to the behavior testing room 30 min prior to beginning the first trial to habituate to its conditions. Animals were familiarized to the apparatus before every recording based on the behavioral test and were subjected to keeping the condition as uniform as possible. Before each trial, the chamber was cleaned with water containing a detergent. The animals’ behavior was videotaped. The behavioral tests were conducted by expert observers blinded to the injury status of the mice. The tests are listed below.

#### 2.11.1. Pole Test (PT)

The PT was performed to detect bradykinesia and motor alterations, as previously described [15]. Briefly, the test consists of a 50-cm-high, gauze-taped pole, and mice are placed with their head upwards. Two parameters were assessed: time necessary for the animals to turned themselves in a downward direction (time to T turn) and time to descend to the base (total time).

#### 2.11.2. Rotarod Test (RT)

Motor coordination was evaluated with a rotary rod apparatus, as previously described [33]. In brief, the mice were placed on the rotating rod (1-cm diameter for mice), and the rotation speed was progressively increased from 0 to 12,000× *g* within 60 s. The latency (i.e., time staying on the rod by a mouse) was recorded. This was repeated (with a rest period that increased by 5 s with each fall) up to five times in one session.

#### 2.11.3. Catalepsy Test

The bar test was used to assess catalepsy, which is characterized as a diminished ability to begin movement and a failure to correct their posture [34]. To examine catalepsy, mice were placed so that their hindquarters were on the bench and their forelimbs on a horizontal bar 4 cm above the bench. The time the mice remained in this position for 30 s or more was recorded.

#### 2.11.4. Elevated Plus-Maze Test (EPM)

The EPM test was used to measure depressive-like behavior, as described previously [35]. The apparatus consisted of two open arms (30 × 5 × 0.25 cm) and two enclosed arms (30 × 5 × 15 cm) in black Plexiglas with a light gray floor, which extended from a central platform (5 × 5 cm); the entire apparatus was elevated by a single central support to a height of 60 cm above the floor level. Briefly, the mouse was placed on the central platform and allowed to explore the maze for 5 min; the number of entries in each arm and the number of crossings were recorded. Anxiety was indicated by a decrease in the percentage of time spent in the open arms and in the number of entries of the open arms.

#### 2.11.5. Open Field Test (OF)

Anxiety-like behavior was monitored by the OF. The apparatus consisted of a white Plexiglas box 50 × 50 cm with its floor divided into 16 squares (four squares were defined as the center and 12 squares along the walls as the periphery). Briefly, each mouse was placed in the center of the box, and the movement was scored as a line crossing when the animal removed all four paws from one square and entered another. The number of crossings and the time spent in the center were calculated and scored for 5 min [36].

#### 2.11.6. Barnes Maze Test (BM)

The BM is a validated test often used to assess spatial learning and memory in rodents [37]. The maze was made from a circular, 13-mm-thick, white PVC slab with a diameter of 48”. Twenty holes with a diameter of 1.75” were made on the perimeter at a distance of 1” from the edge. This circular platform was then mounted on top of a rotating stool 35” above the ground and balanced. All but one of the holes contained a false bottom, while the target hole led to an escape burrow below the maze surface. Briefly, mice were pretrained to enter the escape box by (1) placement into the escape box for 2 min, (2) guidance to the escape box and remaining for 2 min and (3) placement outside the escape box with a glass chamber for up to 3 min and remaining in the escape box for 2 min [38]. After the training session, the performance is measured by the number of errors the rodent makes, and the rate of decline in the number of errors per trial is calculated to represent a learning curve.

### 2.12. Materials

Cypermethrin (Lot# BCCB1117), Diethyl phthalate (Lot #BCBZ8989), Vinclozolin (Lot #BCBZ5052), 17α-ethinyl estradiol (Lot #WXBC9894V), Perfluorooctanesulfonic acid (#0000091932) and Atrazine (Lot# BCBZ3835) were obtained from SIGMA-ALDRICH INC. P.O. (St. Louis, MO, USA). Unless otherwise stated, all compounds were purchased from Sigma-Aldrich. All solutions used for in vivo infusions were prepared using nonpyrogenic saline (0.9% NaCl; Baxter Healthcare Ltd., Thetford, Norfolk, UK).

### 2.13. Statistical Evaluation

All values were expressed as the mean ± standard error of the mean (SEM) of N observations. The images shown were representative of at least 3 experiments performed on diverse experimental days on tissue sections collected from all animals in each group. For the in vivo studies, N represents the number of animals used.

The results involving histology, immunohistochemistry, Western blot and ELISA assay were analyzed by one-way ANOVA, followed by a Bonferroni post hoc test for multiple comparisons. A *p*-value less than 0.05 was considered significant.

## 3. Results

### 3.1. Impact of EDs Exposure on Histopathological Alteration in Midbrain

**Figure 1:**
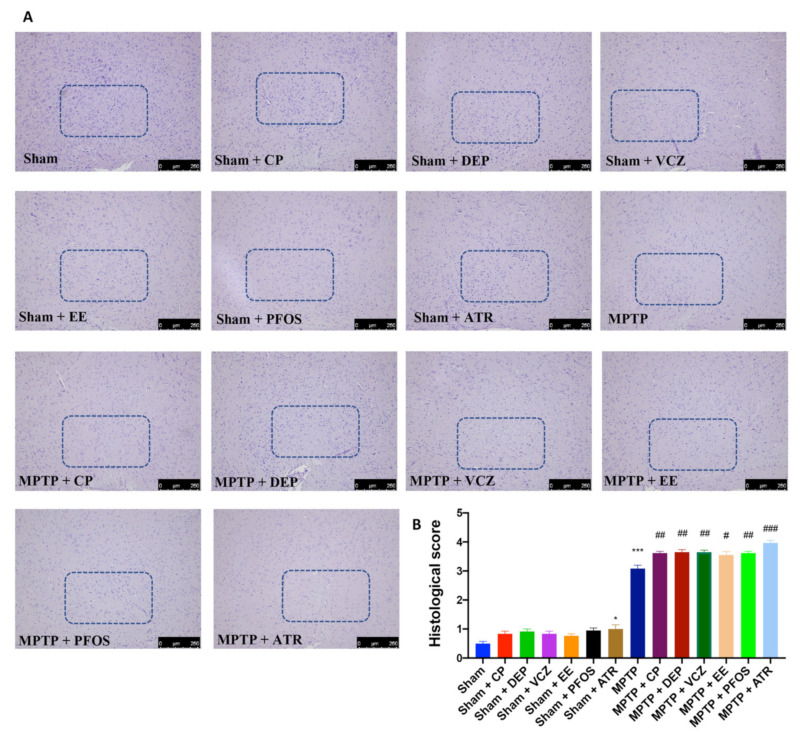
Histological evaluation of the midbrain from all groups (**A**). Histological score (**B**). A 10× magnification is shown (250-µm scale bar). The blue box highlights the area affected by the damage. Data are expressed as the mean ± SEM of N = 6 mice/group. * *p* < 0.05 vs. sham; *** *p* < 0.001 vs. sham; # *p* < 0.05 vs. MPTP; ## *p* < 0.01 vs. MPTP; ### *p* < 0.001 vs. MPTP.

H&E staining was performed to evaluate the histopathological alterations induced by MPTP administration in the midbrain region (Figure 1A,B). Exposure to EDs and the administration of MPTP alone were able to induce alterations in the brain architecture—in particular, nigrostriatal neuronal cell loss—compared to the sham group. Mice treated simultaneously with EDs and MPTP showed greater histological changes than those of the groups treated with single molecules. In particular, ATR exposure showed more evident alterations in the brain architecture compared to other EDs.

### 3.2. Impact of EDs Exposure on DA Metabolism-Related Markers

**Figure 2:**
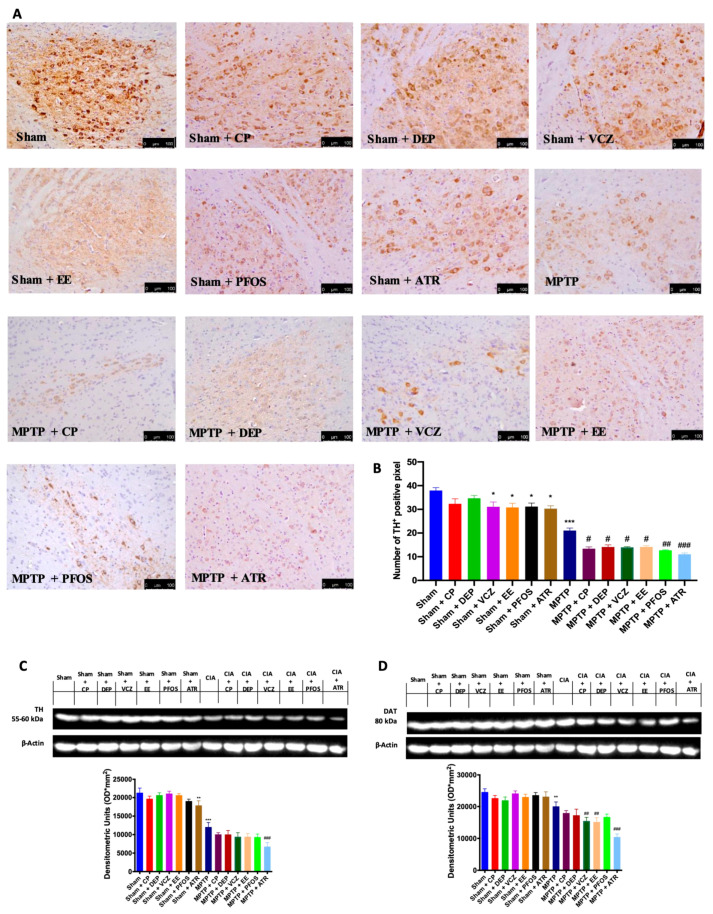
Immunohistochemical evaluation in the midbrain for TH expression (**A**). Graphical quantification of TH expression (**B**). Western blots and densitometric analysis of TH (**C**) and DAT (**D**). A 20× magnification is shown (100-µm scale bar). A demonstrative blot of lysates with a densitometric analysis for all animals is shown. Data are expressed as the mean ± SEM of N = 6 mice/group. * *p* < 0.05 vs. sham; ** *p* < 0.01 vs. sham; *** *p* < 0.001 vs. sham; # *p* < 0.05 vs. MPTP; ## *p* < 0.01 vs. MPTP; ### *p* < 0.001 vs. MPTP.

The cell loss in the midbrain was also reflected in the expression of the dopaminergic-specific markers TH and DAT. The immunohistochemical analysis showed that MPTP injections significantly induce the loss of TH-positive neurons in SN compared to the sham group, while ED exposure in the sham animals caused small alterations in the TH expression (Figure 2A,B). However, exposure to EDs at the same time as MPTP intoxication showed a greater worsening than that of the single molecules (Figure 2A,B). This result was also confirmed by a Western blot analysis (Figure 2C). Additionally, we detected an important reduction of DAT expression in MPTP-injected animals, especially after ED exposure (Figure 2D). We not only observed that exposure to ATR caused an even more significant decrease in both dopaminergic-specific marker expressions.

### 3.3. Impact of EDs Exposure on Endogenous Expression of α-Synuclein (α-Syn)

**Figure 3:**
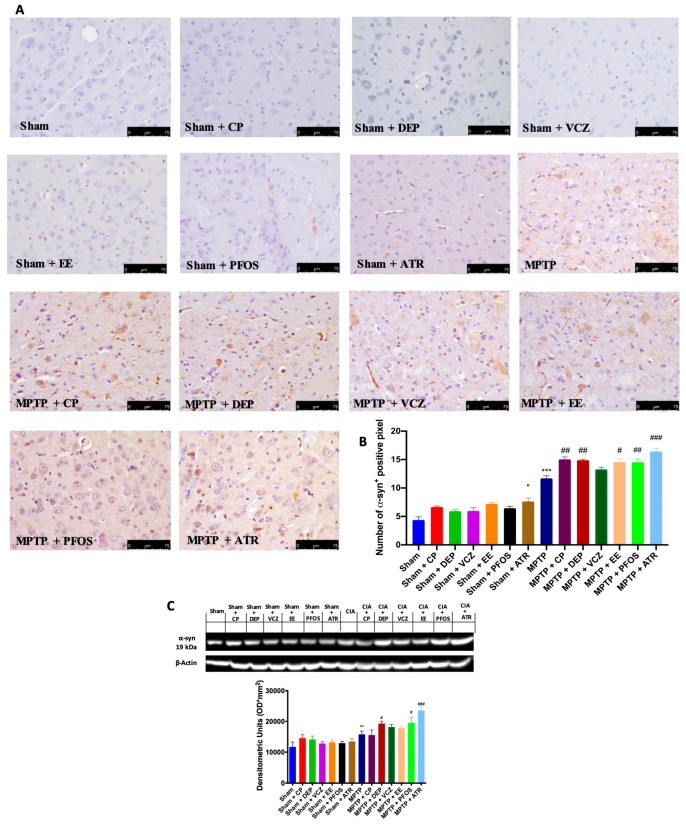
Immunohistochemical evaluation for α-syn expression (**A**). Graphical quantification of α-syn expression (**B**). Western blot and densitometric analysis of α-syn (**C**). A 40× magnification is shown (75-µm scale bar). A demonstrative blot of lysates with a densitometric analysis for all animals is shown. Data are expressed as the mean ± SEM of N = 6 mice/group. * *p* < 0.05 vs. sham; ** *p* < 0.01 vs. sham; *** *p* < 0.001 vs. sham; # *p* < 0.05 vs. MPTP; ## *p* < 0.01 vs. MPTP; ### *p* < 0.001 vs. MPTP.

Since the accumulation of α-syn is a critical indicator of PD, we wanted to evaluate the expression of this protein by immunohistochemical and Western blot analyses. Our results showed a slight increase in α-syn expression in the sham mice after ED exposure, while positive staining for α-syn was significantly higher in the MPTP group compared to sham mice (Figure 3A,B). We not only found that the exposure to ED, particularly to ATR, notably intensified the endogenous expression of α-syn after MPTP intoxication, as also demonstrated by the Western blot analysis (Figure 3C).

### 3.4. Impact of EDs Exposure on Neuroinflammation

**Figure 4:**
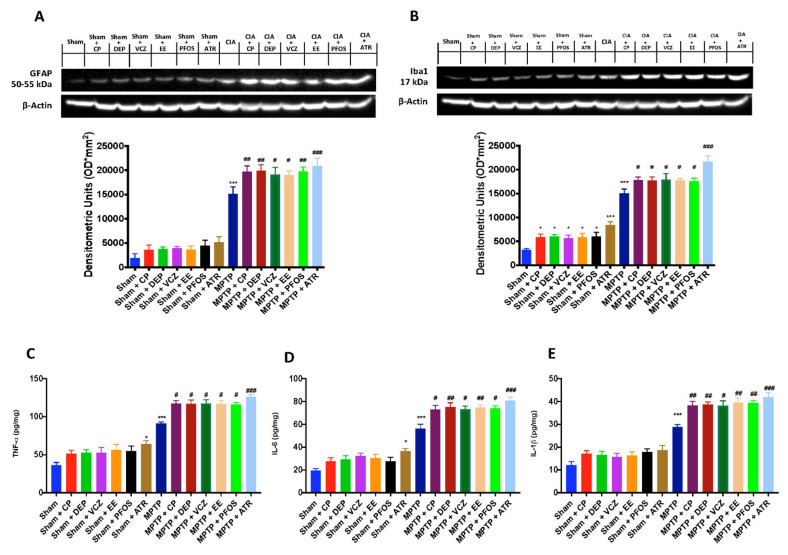
Western blots and densitometric analysis of GFAP (**A**) and Iba1 (**B**). Levels of TNF-α (**C**) IL-6 (**D**) and IL-1β (**E**). A demonstrative blot of lysates with a densitometric analysis for all animals is shown. Data are expressed as mean ± SEM of N = 6 mice/group. * *p* < 0.05 vs. sham; *** *p* < 0.001 vs. sham; # *p* < 0.05 vs. MPTP; ## *p* < 0.01 vs. MPTP; ### *p* < 0.001 vs. MPTP.

To study PD-associated neuroinflammation, we evaluated astrocyte and microglia activation by GFAP and Iba-1 expression, respectively. The levels of GFAP (Figure 4A) and Iba-1 (Figure 4B) were high after ED exposure and MPTP alone compared to the sham group. Eight days after MPTP intoxication, exposure to several EDs and, even more, to ATR caused a major increase in the expression of both proteins.

To confirm neuroinflammation in the midbrain, we analyzed the levels of proinflammatory cytokines. TNF-α (Figure 4C), IL-6 (Figure 4D) and IL-1β (Figure 4E) were significantly upregulated in the MPTP group compared to the sham animals. Cytokines release was more evident after simultaneous MPTP and ED administration and to a more significant extent in the group exposed to ATR.

### 3.5. Impact of EDs Exposure on Nrf-2 Signaling Pathway

**Figure 5:**
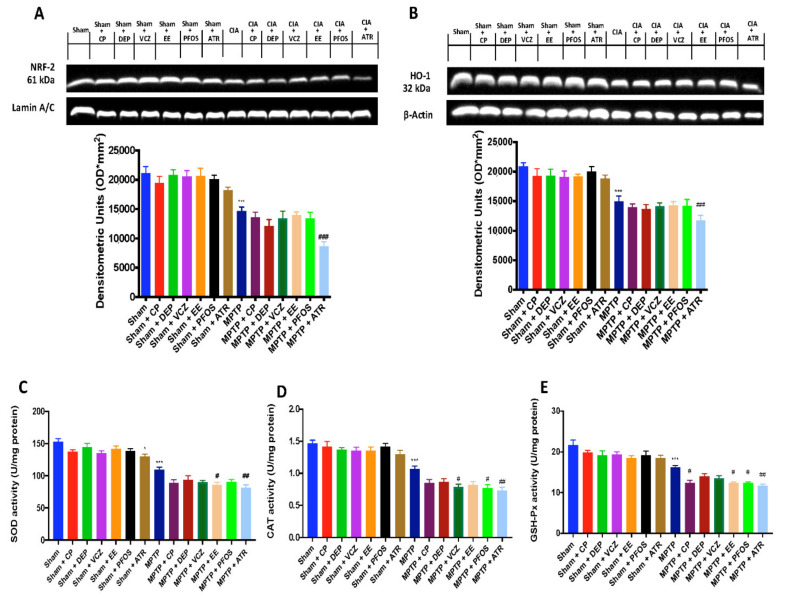
Western blots and densitometric analysis of NRF-2 (**A**) and HO-1 (**B**). Levels of SOD (**C**), CAT (**D**) and GSH-Px (**E**). A demonstrative blot of lysates with a densitometric analysis for all animals is shown. Data are expressed as the mean ± SEM of N = 6 mice/group. * *p* <0.05 vs. sham; *** *p* < 0.001 vs. sham; # *p*< 0.05 vs. MPTP; ## *p* < 0.01 vs. MPTP; ### *p* < 0.001 vs. MPTP.

As is well known, EDs exposure causes cellular toxicity via production ROS. Not only, Nrf2 signaling pathway in involved in PD-associated neuroinflammation. Thus, we evaluated the expression of Nrf-2 (Figure 5A) and HO-1 (Figure 5B) by Western blot analysis and found that these two factors, involved in the physiological regulation of the oxidative balance of the cells, were significantly reduced following MPTP injection, and even more so after simultaneous exposure with EDs.

Additionally, antioxidants such as SOD, CAT and those generated by the GSH system are involved in EDs-induced toxicity. Our results showed an important reduction in levels of SOD (Figure 5C), CAT (Figure 5D) and GSH (Figure 5F) after both exposure to MPTP and EDs, and to a more significant extent in the group expose to ATR.

### 3.6. Impact of EDs Exposure on Oxidative/Nitrosative Stress and DNA Damage

**Figure 6:**
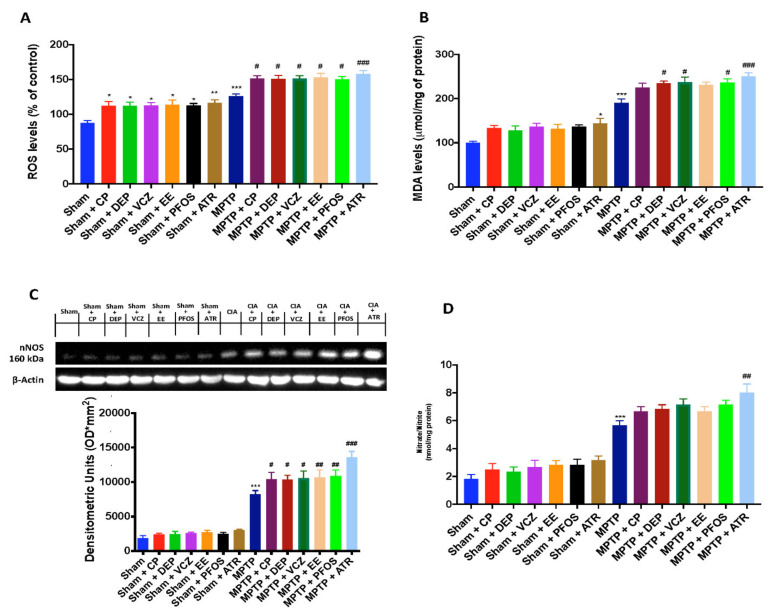
Levels of ROS (**A**) and MDA (**B**). Western blots and densitometric analysis of nNOS (**C**). Nitrate/nitrite levels (**D**). A demonstrative blot of lysates with a densitometric analysis for all animals is shown. Data are expressed as the mean ± SEM of N = 6 mice/group. * *p* <0.05 vs. sham; ** *p* < 0.01 vs. sham; *** *p* < 0.001 vs. sham; # *p* < 0.05 vs. MPTP; ## *p* < 0.01 vs. MPTP; ### *p* < 0.001 vs. MPTP.

The Nrf2 pathway dysregulation caused an increase of oxidative stress and lipid peroxidation, as demonstrated by the ROS levels and MDA activity, respectively. We found that the ROS levels were extremely high after double exposure to MPTP and EDs compared to single molecules (Figure 6A). In the same way, by MDA evaluation, we found a significantly increase in lipid peroxidation after simultaneous exposure to MPTP and the EDs (Figure 6B). Again, ATR-treated mice were more susceptible to oxidative stress and lipid peroxidation compared to the other groups. Oxidative stress is closely linked to nitrosative stress in the neurodegenerative process [39]. In this regard, we investigated nNOS expression following MPTP induction by the Western blot analysis. We found that ED exposure significantly increased the level of nNOS induced by MPTP intoxication, especially in the brain samples collected from ATR-treated mice (Figure 6C). The increased expression of nNOS isoform contributes to the synthesis of nitric oxide (NO), which is rapidly oxidized to nitrite and nitrate [32]. In this study, we measured the nitrate and nitrite levels as the NO index that were significantly increased in the MPTP group compared to the sham groups. Exposure to EDs, particularly to ATR, increased in a more significant manner the NO index (Figure 6D).

### 3.7. Impact of ED Exposure on the MAPK Signaling Pathway

**Figure 7:**
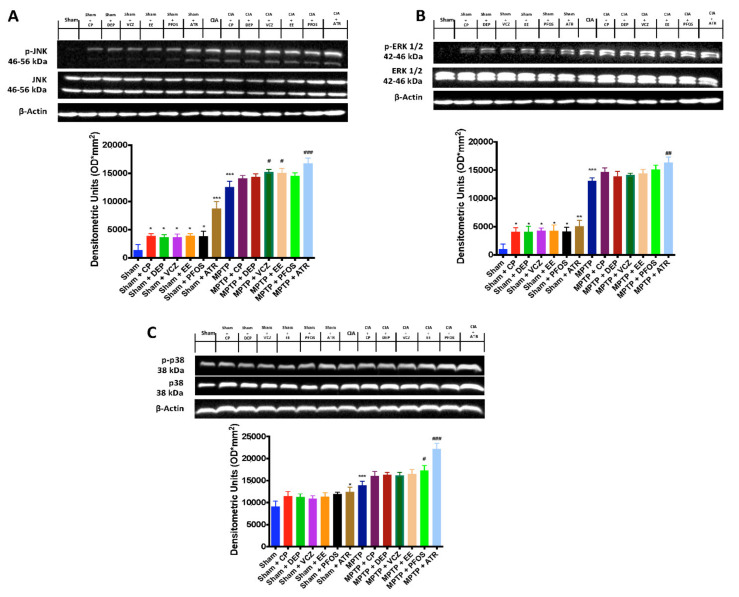
Western blots and densitometric analysis of p-JNK (**A**), p-ERK 1/2 (**B**) and p-p38 (**C**). A demonstrative blot of lysates with a densitometric analysis for all animals is shown. Data are expressed as the mean ± SEM of N = 6 mice/group. * *p* < 0.05 vs. sham; ** *p* < 0.01 vs. sham; *** *p* < 0.001 vs. sham; # *p* < 0.05 vs. MPTP; ## *p* < 0.01 vs. MPTP; ### *p* < 0.001 vs. MPTP.

To investigate the cellular mechanisms that mediate the oxidative stress response, we evaluated the mitogen-activated protein kinase (MAPK) pathway. Our results showed that the activation of MAPK pathways was increased after ED exposure alone and MPTP administration. We also observed that the phosphorylation of JNK (Figure 7A), ERK (Figure 7B) and p38 MAPK (Figure 7C) was more marked in mice subjected simultaneously to both molecules, especially in the group subjected to ATR and MPTP.

### 3.8. Impact of EDs Exposure on Motor Behavioral Impairments

**Figure 8:**
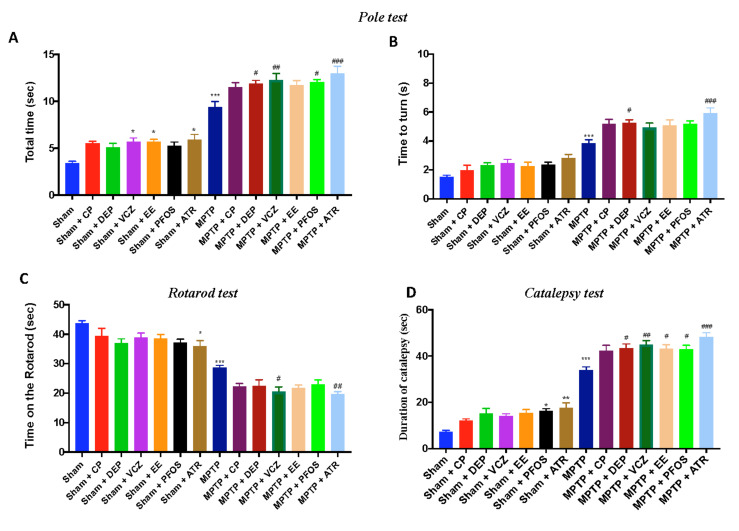
Behavioral analysis motor impairments: Pole test (**A**,**B**). Rotarod test (**C**). Catalepsy test (**D**). Data are expressed as the mean ± SEM of N = 6 mice/group. * *p* <0.05 vs. sham; ** *p* < 0.01 vs. sham; *** *p* < 0.001 vs. sham; # *p*< 0.05 vs. MPTP; ## *p* < 0.01 vs. MPTP; ### *p* < 0.001 vs. MPTP.

Motor functions were assessed using the PT, RT and catalepsy tests. Using PT, the groups subjected to only ED exposure showed minor changes; while after the injection of MPTP, mice exhibited a significant motor dysfunction, as indicated by an increase in the “Time to turn” (Figure 8A) and “Total time” (Figure 7B) spent to descend to the floor. Using a Rotarod apparatus, mice exhibited a significant motor deficit (balance, grip strength and motor coordination) as indicated by a decrease in the time spent on the Rotarod in the MPTP group (Figure 7C). Simultaneous exposure to both MPTP and EDs, especially to ATR, notably increased the motor dysfunction in both tests. The catalepsy test (Figure 7D) confirmed that the ED exposure, especially ATR, increased the behavioral impairment induced by MPTP intoxication.

### 3.9. Impact of EDs Exposure on Depression- and Anxiety-Like Behaviors and Cognitive Alteration

**Figure 9:**
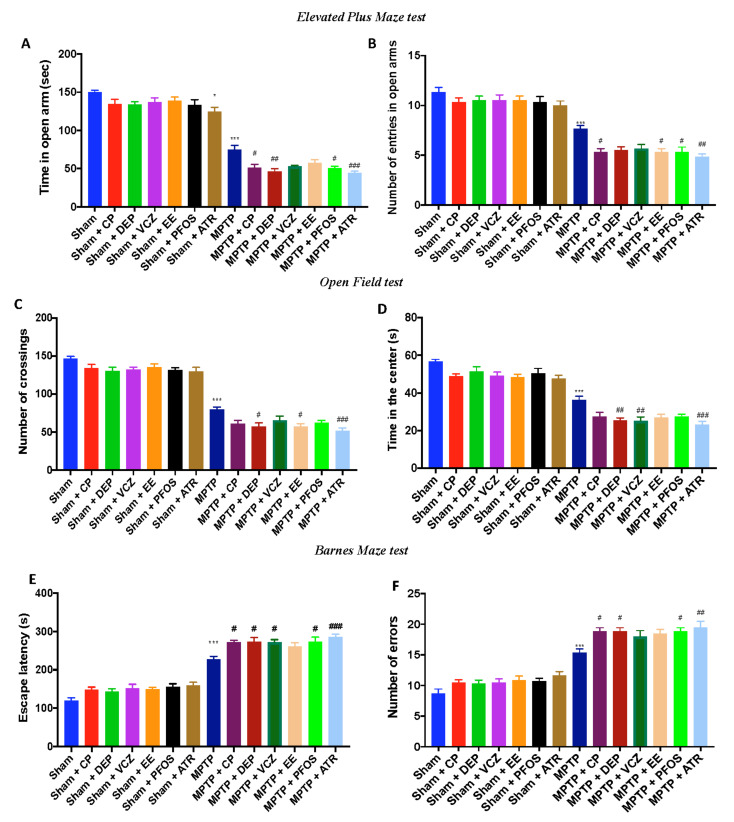
Behavioral analysis for cognitive alteration: Elevated Plus Maze test (**A**,**B**); Open Field test (**C**,**D**); Bernes Maze test (**E**,**F**). Data are expressed as the mean ± SEM of N = 6 mice/group. * *p* < 0.05 vs. sham; *** *p* < 0.001 vs. sham; # *p*< 0.05 vs. MPTP; ## *p* < 0.01 vs. MPTP; ### *p* < 0.001 vs. MPTP.

We performed the EPM and OF to evaluate depressive-like and anxiety-like behaviors in MPTP-treated mice. We noted that mice exposed to EDs without MPTP showed minor behavioral alterations compared to the sham animals. Using the EPM test, the time spent in open arms (Figure 9A) and the number of entries in open arms (Figure 7B) were significantly reduced in all groups after MPTP induction but in a more significant manner in ATR-treated mice, suggesting a depressive-like behavior.

The anxiety condition was observed in mice following MPTP administration using the OF. As expected, mice exposed simultaneously to EDs and MPTP traveled significantly shorter distances (Figure 9C) and showed an increase in the tendency to stay outside the field near the wall (not in the central area, Figure 9D) when compared to MPTP alone. Moreover, ATR showed more significant alterations compared to the other groups.

Furthermore, to evaluate the cognitive alterations, we subjected mice to the BM test. We observed a significant increase in escape latency (Figure 9E) and in the average number of errors (Figure 9F) in the mice exposed to several EDs after MPTP. Again, we found that ATR-treated mice were more susceptible to showing anxiety and depression compared to the other EDs.

## 4. Discussion

EDs have been the subject of rigorous scientific investigation in recent years due to the potential for a variety of adverse health outcomes. This is a difficult problem to solve due to the complexity of the exogenous substance that people are exposed to every day, as EDs are substances found in our environment and in food and consumer products [40]. Currently, the United States Environmental Protection Agency (EPA) and EU have restricted the use of chemicals suspected of causing harm not only to human health but also to farm animals and wildlife, groundwater or to the atmosphere [41,42]. Among EDs, ATR stands out for its wide use globally, with a wide range of applications due to its relatively high efficacy and low price [43]. However, ATR and its metabolites are frequently detected in water, soil or other surfaces, showing long-term residual persistence and chemical stability. Increasing evidence points to ATR as a high-risk pesticide for human health, as it has a fairly long half-life and presents potential ecotoxicity and neurotoxicity [44,45]. Most of the studies on EDs primarily have focused on reproductive and development toxicity; therefore, investigations are needed to demonstrate the impact of ED exposure outside of the reproductive axis and, in particular, on the nervous system to better understand whether it can be correlated with neurodegenerative processes. Thus, this is a relatively new area of research. Some studies have recently demonstrated that exposure to EDs and, in particular, to ATR disrupts the control of the hypothalamic–pituitary–gonadal axis and reduces the level of neurotransmitters in the brain, especially on the dopaminergic system. This can lead to alterations in the DA levels in the nigrostriatal dopaminergic system [21,46].

Considering the above, in this study, we wanted to evaluate the potential negative effects of exposure to several EDs on the dopaminergic system using an experimental model of PD induced by intraperitoneal administration of the neurotoxin MPTP. First of all, we analyzed the alterations of dopamine production with DA metabolism-related markers such as TH, which is the enzyme responsible for catalyzing the conversion of amino acid L-tyrosine into dihydroxyphenylalanine (DOPA), a precursor for dopamine, and DAT, a member of a large family transporters, which control the synaptic activity of released dopamine by rapid reuptake of the neurotransmitter into presynaptic terminals. Our results showed that exposure to EDs significantly raised the loss of TH and DAT induced by the administration of MPTP. We not only found that the alteration of dopamine metabolism was more marked in ATR-treated mice compared to other groups. The altered DA levels represent an impairment in the regulation of this neurotransmitter; accordingly, some of its metabolites accumulate and may exert some neurotoxic and cytotoxic effects within the dopaminergic neurons and mediate mitochondrial dysfunction, which may lead to α-syn aberrant accumulation in the SN [47], which is a crucial marker of PD pathogenesis. Thus, we evaluated the endogenous expression of α-syn, and we observed that this marker significantly increased in the brains taken from mice treated with MPTP compared to the sham groups, while ED exposure induced an even more significant upregulation of α-syn endogenous expression—in particular, in the ATR group. Importantly, EDs do not release the endogenous monomer α-syn directly. However, α-syn aggregation can be considered as a stochastic event, which would increase under conditions of oxidative stress, mitochondrial insufficiency, and neuroinflammation, forming the initial seed nuclei that would escape cellular clearance due to a perturbed proteostasis [48,49,50]. It has been widely demonstrated that increased α-syn expression, as well as exposure to EDs or toxins, promote aggregation [48,51] and, consequently, the formation of Lewy bodies. Therefore, we can hypothesize that the EDs in combination with the induction of MPTP exacerbate the damage and, thus, indirectly induce the upregulation of the endogenous monomer α-syn. Neuroinflammation has been demonstrated to sustain and exacerbate dopaminergic neuronal loss and dopamine deficiency [14,52]. Neuroinflammation is evidenced by the presence of activated microglia and reactive astrocytes in the brain parenchyma, as demonstrated by an important increase in the expression of GFAP and Iba-1 after MPTP intoxication. ATR exposure showed a more marked increase in astrocytic and microglial activation markers compared to the exposure of other EDs, confirming the importance of neuroinflammation in neurodegenerative processes. The Nrf2 signaling pathway has an important effect on the modulation of neuroinflammation and on the regulation of cellular defense enzymes against oxidative stress [53]. On the other hand, oxidative stress has emerged as an investigative mechanism of ED toxicity. ATR and the other EDs appear to impair the oxidative homeostasis via direct or indirect mechanisms, including the increase of oxidative mediators and reduction of antioxidant enzymes, determining alterations in the cell signaling pathways and production of ROS, contributing to ED organ toxicity [44,54,55]. Interestingly, although oxidative stress and ED exposure have often been associated with each other, only a few studies have simultaneously examined the oxidative stress pathways that mediate the impact of ED exposure on neurodegenerative disorders. In this study, we investigated how exposure to EDs contributed to worsening oxidative stress induced by MPTP administration. ATR, even more than other EDs, increased the oxidative stress and ROS and perturbed the Nrf2 signaling pathway in MPTP-intoxicated mice. Our Western blot analysis showed that the induction of PD caused a dysregulation of the Nrf2 signaling pathway, as demonstrated by a decrease in the nuclear expression of Nrf2. We observed that the Nrf2 levels were more significantly decreased in ATR-treated rats compared to the other groups. The loss of Nrf2-mediated transcription increases the vulnerability of dopaminergic neurons to oxidative stress. Under normal conditions, Nrf2 is inhibited in the cytoplasm by the binding with Keap1. However, under oxidative stress conditions, the dissociation of Keap1 disrupts its interaction with Nrf2, resulting in the binding of Nrf2 to the promoter regions of ARE-mediated antioxidant genes, which induces the transcription of phase II detoxifying and antioxidant enzymes such as HO-1 and Mn-SOD [56]. Consequently, the reduction of antioxidant enzymes was also detected after simultaneous exposure to EDs and MPTP. Due to an imbalance of the antioxidant/oxidant homeostasis, ROS are excessively produced and still cause lipid peroxidation and nitrosative stress, as demonstrated by elevated MDA, nNOS and NOx levels. Thus, we postulated that, when ROS are excessively produced, antioxidants become overloaded, and their activity begins to decrease. This could at least partially explain the mechanism of ED toxicity. Moreover, emerging evidence suggests that the oxidative stress response is mediated by the activation of a cascade of MAPKs [10]. The formation of ROS activates JNK and p38 MAPK, which can lead to an increase in ROS production. Too much ROS can, in turn, affect the activation of p38MAPK, the formation of a feedback loop plays an important role in the development of PD [57]. Therefore, the proteins of the MAPK family were measured to identify the underlying molecular and cellular mechanisms of the toxicity of EDs and increased the oxidative stress. In this study, we found that MPTP induces the activation of the p-JNK, p-p38 and p-ERK signaling pathways, whereas, in the brain samples of mice exposed to Eds, the expression of these proteins was increased more significantly after exposure to ATR.

Finally, we performed behavioral tests to demonstrate that the alterations in the dopaminergic system also reflected the behavioral alterations. First, we analyzed the changes of the motor functions, by the PT, RT and catalepsy tests. Animals exhibited considerable motor deficits revealed by an increased bradykinesia and motor coordination, following simultaneous MPTP and ED administration. Although recognized as the most widespread neurodegenerative motor pathology, PD is accompanied by several nonmotor symptoms that can lead to cognitive and psychiatric disorders [22]. Our behavioral tests showed that mice exposed simultaneously to ED and MPTP exhibited markedly depressive and anxiety-like behavior. The behavioral tests showed that exposure to ATR caused a more pronounced motor dysfunction, as well as a worsening of cognitive alterations.

In summary, our data suggested that ED exposure induces microglia activation, that, in turn, exacerbate oxidative stress and reduce the DA levels in the progression and pathogenesis of neurodegenerative disorders such as Parkinson’s disease. The cellular and molecular mechanisms that underlie the ED exposure-induced neuroinflammation and neurotoxicity is probably due to dysregulation of the Nrf2 signaling pathway, inducing an imbalance in the cellular redox homeostasis. Further studies will be required to clarify whether the Nrf2 signaling pathway will offer new strategies to prevent antioxidant stress during neurodegenerative processes and to investigate the mechanisms by which ED regulates other signaling pathways.

## 5. Conclusions

EDs are a global and ubiquitous problem. The number of EDs has markedly increased over the past 60 years. Humans are constantly exposed to hundreds of EDs mainly through the air, water and food. Identifying the risk associated with a single ED is complex, because humans are exposed to low doses of hundreds of chemicals starting at the fetal stage of life. A strategy for the prevention of exposure to EDs is urgently needed. Several agencies (e.g., US EPA and European Food Safety Agency) are regulating EDs to limit their exposure. Additional studies are required to understand the potential mechanisms of ecotoxicity and neurotoxicity and determine the potential health risks associated with exposure to EDs.

## Data Availability

The datasets used in the current study are available from the corresponding author (rfusco@unime.it) upon reasonable request.
