# Correction: D’Amico et al. Toxic Exposure to Endocrine Disruptors Worsens Parkinson’s Disease Progression through NRF2/HO-1 Alteration. *Biomedicines* 2022, *10*, 1073

**DOI:** 10.3390/biomedicines12040834

**Published:** 2024-04-10

**Authors:** Ramona D’Amico, Enrico Gugliandolo, Rosalba Siracusa, Marika Cordaro, Tiziana Genovese, Alessio Filippo Peritore, Rosalia Crupi, Livia Interdonato, Davide Di Paola, Salvatore Cuzzocrea, Roberta Fusco, Daniela Impellizzeri, Rosanna Di Paola

**Affiliations:** 1 Department of Chemical, Biological, Pharmaceutical and Environmental Sciences, University of Messina, 98166 Messina, Italy; rdamico@unime.it (R.D.); rsiracusa@unime.it (R.S.); tiziana.genovese@unime.it (T.G.); aperitore@unime.it (A.F.P.); interdonatol@unime.it (L.I.); dipaolad@unime.it (D.D.P.); dimpellizzeri@unime.it (D.I.); 2 Department of Veterinary Science, University of Messina, 98168 Messina, Italy; egugliaandolo@unime.it (E.G.); rcrupi@unime.it (R.C.); dipaolar@unime.it (R.D.P.); 3 Department of Biomedical, Dental and Morphological and Functional Imaging, University of Messina, 98125 Messina, Italy; marika.cordaro@unime.it; 4 Department of Clinical and Experimental Medicine, University of Messina, 98125 Messina, Italy

**Errors in Figures**

In the original publication [1] for panels in Figure 2 and Figure 3, the authors unintentionally assembled some wrong figures. In Figure 2, in the group MPTP + PFOS, they inadvertently attached the wrong picture, and in Figure 3, in the groups Sham + CP and Sham + DEP, they made a copy and paste of the same figure. The authors checked all the data in their laboratory, and they were able to find the original photos and prepare a revised figure using an appropriate representative image from their database belonging to the experimental groups in question. The authors apologize for any inconvenience caused by this oversight. These errors do not affect the results and conclusions published in the article. The new Figure 2 and Figure 3 appear below. The authors state that the scientific conclusions are unaffected. This correction was approved by the Academic Editor. The original publication has also been updated.

## Figures and Tables

**Figure 2:**
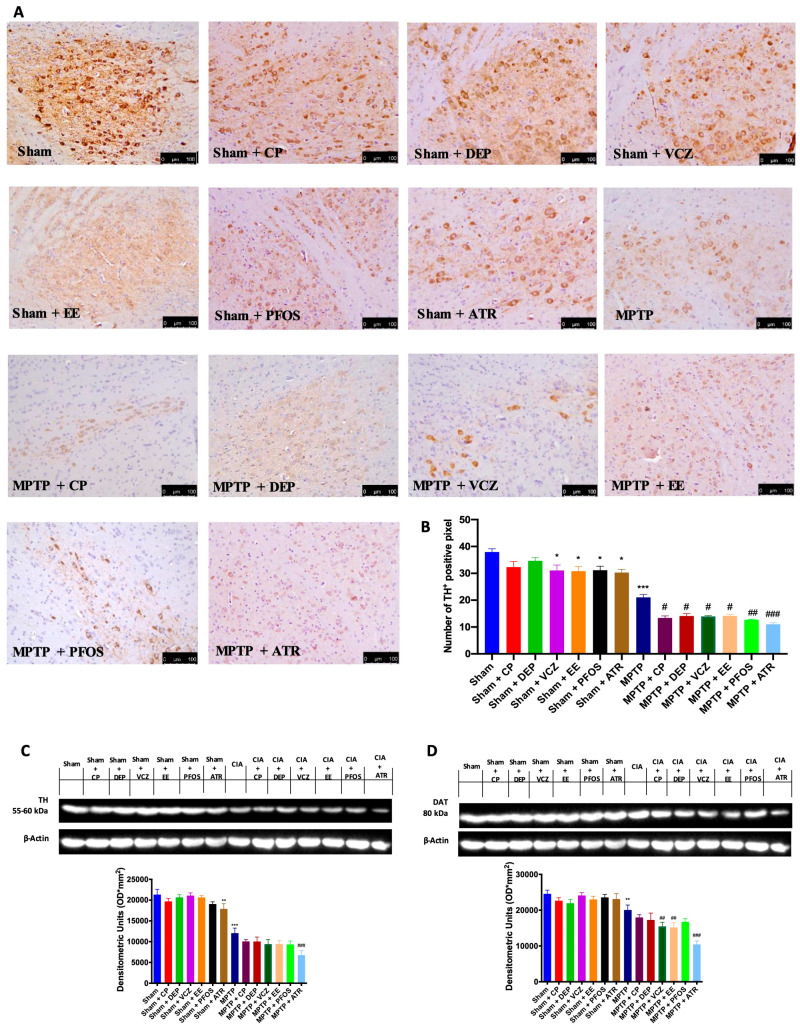
Immunohistochemical evaluation in the midbrain for TH expression (**A**). Graphical quantification of TH expression (**B**). Western blots and densitometric analysis of TH (**C**) and DAT (**D**). A 20× magnification is shown (100-µm scale bar). A demonstrative blot of lysates with a densitometric analysis for all animals is shown. Data are expressed as the mean ± SEM of N = 6 mice/group. * *p* < 0.05 vs. sham; ** *p* < 0.01 vs. sham; *** *p* < 0.001 vs. sham; # *p* < 0.05 vs. MPTP; ## *p* < 0.01 vs. MPTP; ### *p* < 0.001 vs. MPTP.

**Figure 3:**
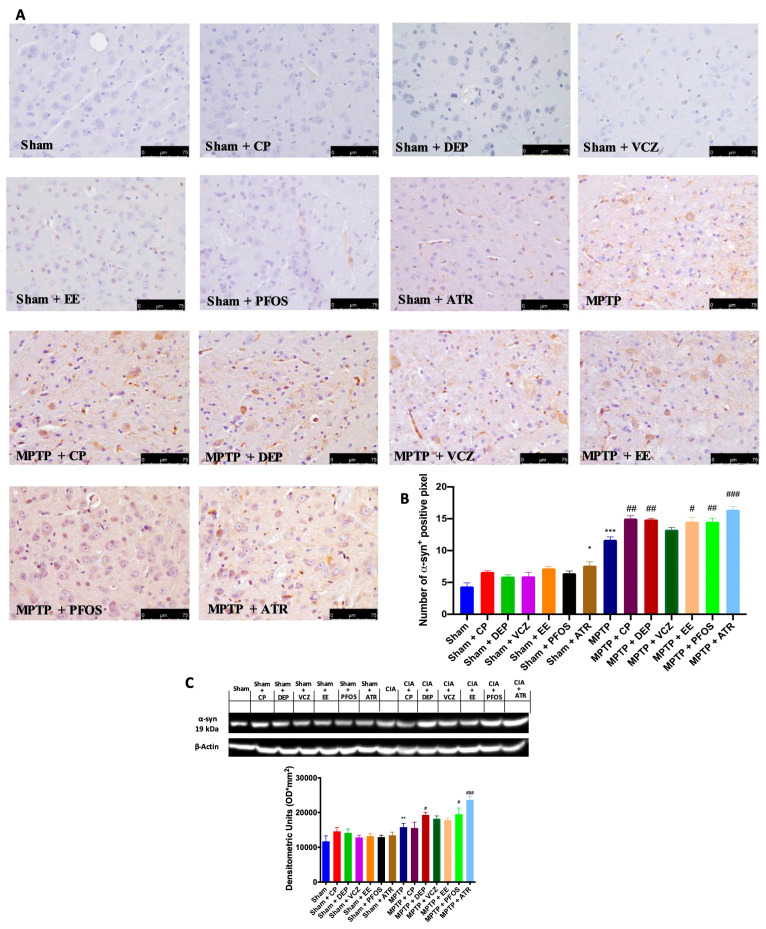
Immunohistochemical evaluation for α-syn expression (**A**). Graphical quantification of α-syn expression (**B**). Western blot and densitometric analysis of α-syn (**C**). A 40× magnification is shown (75-µm scale bar). A demonstrative blot of lysates with a densitometric analysis for all animals is shown. Data are expressed as the mean ± SEM of N = 6 mice/group. * *p* < 0.05 vs. sham; ** *p* < 0.01 vs. sham; *** *p* < 0.001 vs. sham; # *p* < 0.05 vs. MPTP; ## *p* < 0.01 vs. MPTP; ### *p* < 0.001 vs. MPTP.

## References

[1] D’Amico R., Gugliandolo E., Siracusa R., Cordaro M., Genovese T., Peritore A.F., Crupi R., Interdonato L., Di Paola D., Cuzzocrea S. (2022). Toxic Exposure to Endocrine Disruptors Worsens Parkinson’s Disease Progression through NRF2/HO-1 Alteration. Biomedicines.

